# Medaka, *Oryzias latipes*, egg envelopes are created by ovarian-expressed ZP proteins and liver-expressed choriogenins

**DOI:** 10.1186/s40851-022-00194-2

**Published:** 2022-07-28

**Authors:** Devun S. Birk, Shinji Onose, Masato Kinoshita, Kenji Murata

**Affiliations:** 1 University of California, Davis. Center for Health and the Environment, Davis, CA 95616 USA; 2 Fordays Co., Ltd, Tokyo, 103-0016 Japan; 3 Division of Applied Biosciences, Graduate School of Agriculture, Kyoto University, Kyoto, 606-8502 Japan

**Keywords:** Choriogenins, Chorion, Chorion formation, Egg envelope, Medaka, Oogenesis, Zona pellucida, ZP protein

## Abstract

The medaka (*Oryzias latipes*) egg envelope (chorion) is composed of three major glycoproteins, Zona Interna (ZI)-1, -2, and -3, that originate in the spawning female liver as the precursor proteins Choriogenin (Chg.)H, Chg.Hm, and Chg.L, respectively. These ZI and Chg. proteins contain a structural ZP protein domain that is conserved among the egg envelope proteins of all animals. While ovarian expression of ZP proteins (e.g., ZPCs and ZPB) has been reported in medakas, the functions of these proteins remain unknown. Thus, the present study aimed to determine whether the ovary-expressed medaka ZP protein, mZPC5, is involved in forming the chorion matrix.

The mZPC5 gene (*mzpc5*) was expressed in the ovaries but not the livers of mature female medakas, as shown by reverse transcription-polymerase chain reaction assays with *mzpc5-*specific primers. In situ hybridization analysis revealed that ovarian *mzpc5* expression was restricted to the ooplasm of early (stage I–III) previtellogenic oocytes, and its expression signal weakened with oocyte growth. Following sodium-dodecyl-sulfate polyacrylamide gel electrophoresis (SDS-PAGE) and Western blotting analysis with anti-mZPC5 antibodies, two immunoreactive proteins were detected in the ovary and chorion extracts. These proteins were approximately 50 and 74 kDa in size, like ZI-3 and ZI-2, respectively.

Immunohistochemical assays using anti-mZPC5 and anti-Chg.H antibodies localized the mZPC5 protein in the ooplasm of early previtellogenic oocytes. With oocyte growth, mZPC5 tended to accumulate in the chorion, co-localizing with Chg.H.

We previously showed that ovary-expressed ZP proteins could not compensate for Chg.L function loss in gene knock-out (*chg.l -/-*) medakas. As in our previous study, the *chg.l-/-* females produced oocytes with thin chorions, resulting in infertile soft eggs. However, in the present study, mZPC5 and Chg.H were co-localized in the *chg.l-/-* chorions. These results suggested that in the medaka previtellogenic oocyte, 1) mZPC5 is secreted from the ooplasm and deposited on the outer surface of its plasma membrane, creating the thin chorion layer; and 2) following the accumulation of liver-derived Chgs., the 3D structure of the chorion matrix is formed cooperatively with mZPC5 and Chgs. during oogenesis. More research is needed to confirm the functions of mZPC5 in chorion structure and physiology.

**Supplementary Information:**

The online version contains supplementary material available at 10.1186/s40851-022-00194-2.

## Background

The egg envelope (chorion, zona radiata) in fish is an extracellular matrix that protects the oocytes and embryos from physical, chemical, and other detrimental environmental factors. The egg envelope is called the “vitelline membrane” in amphibians, the “perivitelline membrane” in birds, and the “zona pellucida” (ZP) in mammals [1]. During the cortical reaction of fertilization, the egg envelope changes in structure and forms the fertilization membrane. In fish, alveoline [2] and transglutaminase [3–5] are released from cortical granules, promoting hardening of the chorion by affecting the cross-linkage between the protein subunit molecules. At hatching, the major glycoproteins of the inner layer of the chorion are targeted by the hatching enzyme [6].

**Fig. 1:**
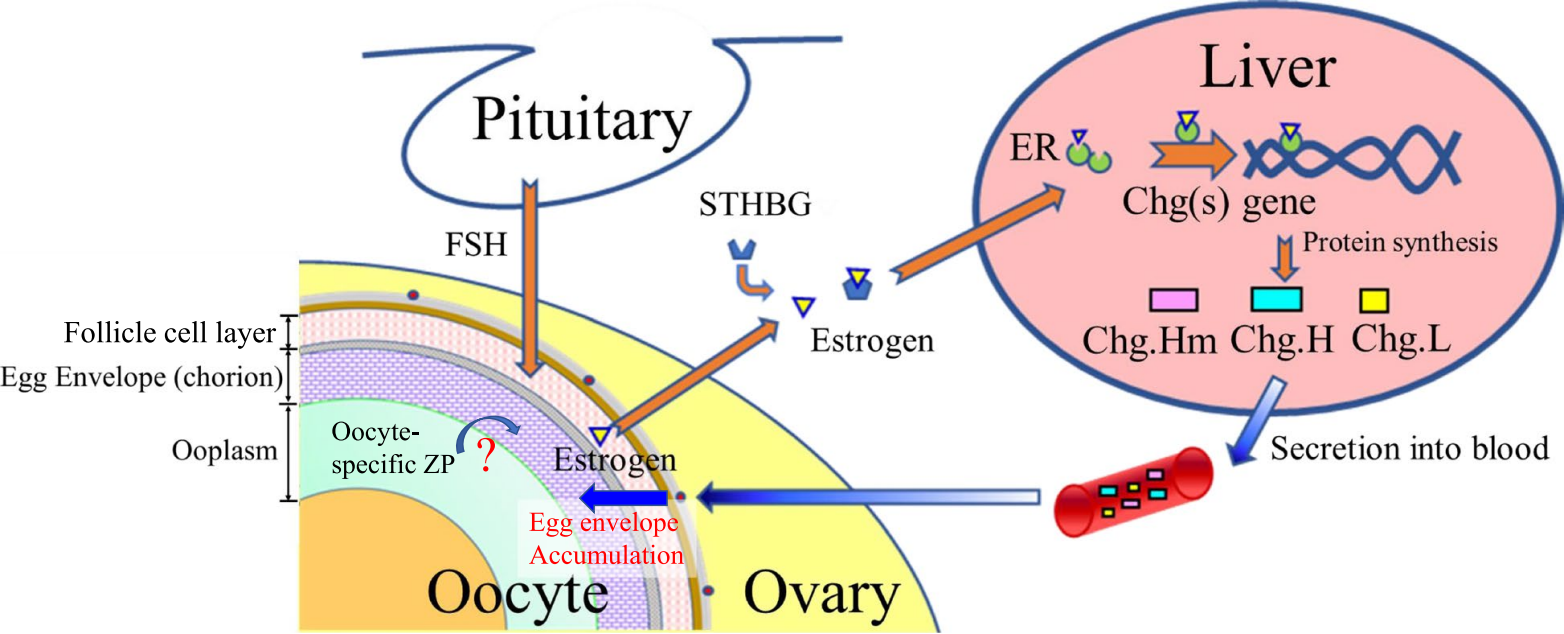
Schematic illustration of the pituitary-ovary-liver axis for medaka, *Oryzias latipes*, choriogenesis. Choriogenesis, i.e., chorion formation, includes hepatic expression of genes for chorion components (e.g., structural proteins), transfer of liver-derived proteins into the ovary, and accumulation of the proteins to form the chorions of developing oocytes in the ovary. Abbreviations: Chg(s). – choriogenin(s); ER – estrogen receptor; FSH – follicle-stimulating hormone; STHBG – sex steroid hormone-binding globulin; ZP – zona pellucida (mammalian egg envelope). “Oocyte-specific ZP?” pertains to the question the present study aimed to address since it was previously unknown whether ovarian ZP proteins localize in the medaka chorion or play a role in its function/structure

Generally, the fish chorion consists of two or three structurally different layers. In the eggs of medaka, *Oryzias latipes*, the envelope is usually divided into two layers: a thin outer layer and a thick inner layer [7]. The major portion of the chorion is composed of the inner layers, consisting of three major glycoproteins: Zona Interna (ZI)-1, -2, and -3 [8]. These mature ZI glycoproteins are formed by the conversion of precursor proteins originating in extra-chorionic tissues. Two different sites have been identified as sites synthesizing the fish chorion precursor proteins. These sites include the liver and oocytes of spawning females. In 1984, Hamazaki et al. [9] were the first to report the possibility that one of the chorionic glycoproteins was produced in the liver of spawning females. Since the publication by Hamazaki et al. [9], additional biochemical and immunochemical data have revealed that all of the major chorionic glycoproteins are synthesized in the liver of spawning females as the yolk precursor protein “vitellogenin” [8–15] upon induction by estrogen (Fig. 1).

Previous studies have expanded our understanding regarding such liver-derived precursor proteins in medakas. The accumulation of a low (49-kilodalton (kDa))-molecular-weight (MW) chorion precursor protein was initially identified by injecting radiolabeled molecules into the abdominal cavities of mature female medakas [16]. Later, the genes encoding medaka chorionic glycoproteins were cloned from a complementary deoxyribonucleic acid (cDNA) library of sexually mature females’ livers [17–19]. These genes were designated Choriogenin H (*chg.h*), Choriogenin H minor (*chg.hm*), and Choriogenin L (*chg.l*) (Fig. 1).

The predicted amino acid sequences of the Chg. genes encode the ZP domain that is conserved among egg envelope-related genes in all animals [20, 21]. The ZP domain is a portion of the protein polypeptide chain that is self-stabilizing, folds independently from the rest of the chain, and contributes to the general role of the protein as it relates to the structure and function of the egg envelope. Thus, the liver-derived Chg.H, Chg.Hm, and Chg.L precursor proteins that form the mature ZI-1, ZI-2, and ZI-3 proteins, respectively, in the chorions of medakas (Fig. 1), are homologous to ZP proteins in mammals. Following the nomenclature of Spargo and Hope [22], ZI-1 and -2 correspond to ZPB, and ZI-3 corresponds to ZPC. This terminology was used in the present paper for fish ZP genes.

As liver-expressed chorionic glycoproteins are not restricted to medakas, cDNAs encoding such proteins were cloned from a cDNA library based on liver tissues from sexually mature female winter flounder [23], rainbow trout, and Atlantic salmon [24]. However, the synthesis of the chorionic glycoproteins seems to be restricted to the oocyte in goldfish, carp, and zebrafish [25–28].

While the production of ZP proteins in the ovaries of medakas has been reported [28, 29], the lack of biochemical and immunochemical data makes it challenging to identify their functions. This latter point is especially true regarding whether the ZP proteins originating in the medaka ovaries are components of the chorions. Kanamori and colleagues [29, 30] reported that ZP-domain-containing proteins were expressed in the medaka ovary. They named the proteins ZPC1 ~ ZPC5 because 1) their predicted amino acid sequences were similar to those of mammalian ZPC; and 2) their gene structure suggested their regulation was controlled by basic helix-loop-helix transcription factors [29, 30]. Helix-loop-helix transcription factors are dimeric proteins involved in transcribing DNA to ribonucleic acid (RNA), i.e., transcription. They are found in almost all eukaryotes, and in animals, they are essential regulators of embryonic development. While the Figα helix-loop-helix transcription factor is thought to be critical in regulating ZP gene transcription, *chg.h*, *chg.hm*, and *chg.l* expression is induced by estrogen in the liver of spawning female, and sometimes adult male, medakas. In 2012, Hirakawa et al. [31] reported that in medaka testis-ova (the occurrence of oocytes in the testis of male fish), the expression of *zpc5*, rather than other oocyte-specific ZP genes, was strongly upregulated by exposure to estrogen; thus, *zpc* may be a marker for monitoring the abnormal condition. However, the function of the gene products of these oocyte-specific ZPs and whether they are components of the chorion remained unknown.

In 2022, it was reported [32] that *chg.l-/-*, i.e., homologous Chg.L-knockout (KO), females, produced very thin chorions and spawned string-like materials containing “smashed eggs.” In these females, the gene products of ovary-expressed ZPCs failed to compensate for the loss of Chg.L function in forming the correct extracellular chorion matrix [32]. We hypothesize that during medaka choriogenesis, some of the ovary-expressed ZP proteins also contribute to forming the chorion matrix that interacts with Chg,H, Chg.Hm, and Chg.L. In a first step to identifying the functions of the ovary-expressed ZP proteins (e.g., ZPCs), mZPC5 was selected to determine its gene expression pattern and location in the ovary.

## Materials and Methods

### Fish and tissues

An orange medaka variety [33] was maintained in an aquarium, with recirculating water at 26 °C and a 14-/10-h day/night cycle, in the University of California Davis (UC Davis) medaka facility. The fish were handled according to an approved institutional animal care protocol (UC Davis protocol #22,463) and anesthetized with 0.03% tricaine methanesulfonate (MS-222) before tissue dissection. Following procedures described by Murata et al. [12], blood, ovary (mature females only), and liver tissue samples were extracted from mature female and male medakas (n ≥ 5 per sex) at > 12 weeks of age to assess the expression of *mzpc5* and *chgs.* and the tissue immunoreactivity against anti-mZPC5 and anti-Chg.H antibodies. The maximum amount of blood was collected from each fish for biochemical and histological analysis by cutting and bleeding it from the caudalis. Briefly, after anesthesia, the body was wiped to removed excess water. Then the tail was cut off with surgical eye scissors at a position of two-thirds of the distance between the genital pore and the start of the tail fin (Supplement Fig. 1). Each blood sample was collected by soaking the cut end of the body for 20 min (min) in a 1.5-mL tube containing ice-cold phosphate-buffered saline (PBS) with 0.4 mg/ml phenylmethylsulfonyl fluoride (a serine protease inhibitor commonly used in the preparation of cell lysates) and 40 mM ethylendiamine tetraacetate (EDTA; pH = 7.2). This procedure prevented extrahepatic tissue contamination by liver-derived Chgs. released into the blood.

Developmental stages of ovarian oocytes followed and were determined as described by Iwamatsu et al. [34].

### The *chg.l -/-* transgenic medaka strain

The *chg.l-/-* transgenic strain was established using transcription activator-like effector nuclease (TALEN) restriction enzymes [35] and detailed procedures described by Murata and Kinoshita [32]. As reported previously [32], the *chg.l*-KO (*chg.l -/-*) female medakas produced oocytes with very thin chorions and spawned string-like material containing “smashed eggs.” At that time, we determined that the gene product of ovary-expressed ZP-domain-containing proteins could not compensate for the loss of Chg.L function in the chorion or support the architecture of the chorion [32].

### Reverse transcription-polymerase chain reaction and cloning of cDNA containing the predicted full-length amino acid sequence of mZPC5

**Fig. 2:**
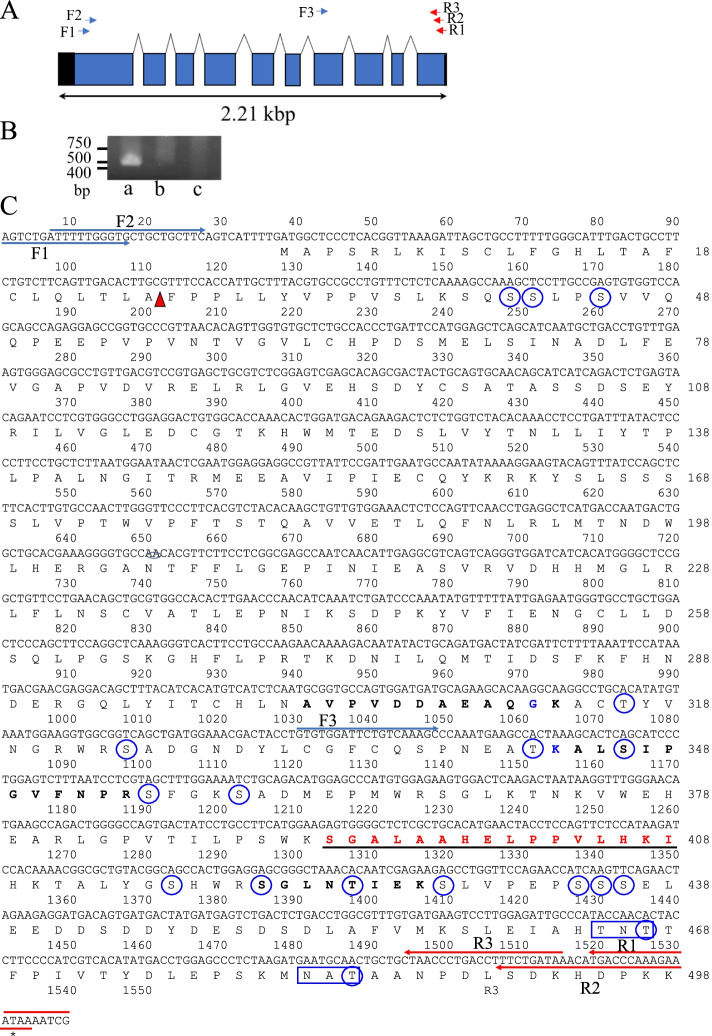
Cloned cDNA encoding mZPC5 in the ovaries and livers of spawning medakas. **A:** the genomic structure of *mzpc5* and the primer position used to identify *mzpc5* expression and clone full-length cDNA. The diagrammed pattern of the genomic structure is based on information from the Ensemble Project database [36]. The blue and red arrows indicate the positions of forward (F1, F2, F3) and reverse (R1, R2, and R3) primers, respectively, that were used for RT-PCR. **B**: the result of the RT-PCR analysis. Ba: RNA obtained from the spawning female ovary. Bb: RNA obtained from the spawning female liver. Bc: RNA obtained from male liver. **C**: the nucleotide and predicted amino acid sequences of newly cloned *mzpc5* cDNA in this study. A one-letter symbol represents each amino acid as defined by the International Union of Pure and Applied Chemistry (IUPAC). The red arrowhead points to the signal sequence cleavage site analyzed using PSORT [37]. Underlined amino acid sequences with bold red letters represent the antigen peptide sequence. The blue and red arrows indicate the locations of primers used for RT-PCR analysis. Blue-circled letters indicate the predicted amino acids connecting to O-linked oligosaccharides [38]. Letters boxed in blue represent the predicted peptide sequence at the N-glycosylation site [39]. Bold blue amino acids (^312^G and ^343^ K) differ from the amino acids in ZPC5 (accession # AAN31192). The amino acid sequences of the peptides shown in bold black letters were obtained from the immunoreactive proteins (~74 kDa) in Figs. 4b and 5b following MS/MS analysis

RNA was extracted from the ovaries and livers of spawning females and the livers of mature males according to manufacturer’s instructions provided in the RNeasy Plus Mini Kit (QIAGEN, Redwood City, CA 94,063). Reverse transcription-polymerase chain reaction (RT-PCR) assays were performed with specific primer sets following instructions in the Titanium® One-Step RT-PCR Kit (Takara Bio USA, Inc. Mountain View, CA 94,043). Primer set 1 consisted of *mZPC5*F3 (5′-GTGTGGATTCTGTCAAAGC-3′) and *mZPC5*R3 (5′-TTATCAGAAAGGTCAGGGTTAG-3′) (Figs. 2A and 2B), and primer set 2 consisted of *mZPC5*F1(5′-AGTCTGATTTTTGGGTGCTGCT-3′) and *mZPC5*R1 (5′-GCATTTTATTTCTTTGGGTCATGTTTATCA-3′). Nested PCR was performed using primer set 3 [*mZPC5*F2 (5′-ATTTTTGGGTGCTGCTGCTTCA-3′) and *mZPC5*R2 (5′-TTATTTCTTTGGGTCATGTTTATCAGAAA-3′); Fig. 2A and C], and the PCR product amplified by primer set 3. The resulting DNA fragment was inserted into pGEM®-T Easy vector (Promega, Chicago, IL, USA) for sequencing at the UC Davis College of Biological Sciences DNA Sequencing Facility. The genomic structure of *mzpc5* was predicted based on an analysis using the Ensemble Project database [36] and *mzpc5* cloned in this study with primer set 2 (Fig. 2A and C). The signal sequence cleavage site was analyzed using the PSORT computer program [37] to predict the protein sorting, signals, and localization sites in the amino acid sequences. The predicted amino acids connecting to O-linked oligosaccharides and the predicted peptide sequence at the N-glycosylation site were analyzed using the NetOGlyc (version 4.0) [38] and NetNGlyc-1.0 [38] computer programs.

### In situ hybridization

The ovaries were collected and fixed during necropsy (day 1) after cutting and bleeding randomly selected, sexually mature female fish from the caudalis. Tissue fixation was performed overnight at 4 °C using 4% paraformaldehyde in 0.85 × PBS (pH = 7.2), with gentle rotation from a Clay Adams Nutator 1105 Single-Speed Orbital Mixer (12 revolutions/minute (rpm); Becton Dickinson Primary Care Diagnostics, MD USA). The next day, the ovaries were dehydrated with a graded (25%, 50%, 75%, 90%, and 100%) ethanol series for 1 h at each concentration and transferred to fresh absolute ethanol overnight at 4 °C. On day 3, the tissues were placed in 1:1 ethanol and xylene for 1 h at 4 °C and moved to fresh xylene for each of three 1-h treatments at room temperature before embedment in paraffin at 52 °C. The embedded tissues were sectioned to 5-µm thickness using a microtome (LEICARM 2155) (Leica Biosystems, Vista, CA, USA) at the CAMI core facility at the UC Davis Center for Health and the Environment. The prepared sections were deparaffinized in fresh xylene for three separate 10-min (min) soaks followed by 100%, 90%, and 80% ethanol for 15 s (sec) each. This step was followed by three 15-min rinses with 0.1 M fresh phosphate buffer (PB; pH = 7.4) in a Coplin glass horizontal staining jar, using gentle vibration from a Corning PC320 stirrer (Corning, NY USA) on a low-speed setting. The sections were then treated in the following order before being dried.

- 1. proteinase K (1 µg/ml in 10 mM Tris–HCl; pH = 8.0 with 1 mM EDTA) for 2 min,
- 2. 4% paraformaldehyde in PB for 10 min,
- 3. PB for 1 min,
- 4. 0.2 M HCl for 10 min,
- 5. PB for 1 min,
- 6. 0.1 M triethanolamine-HCl (TEA; pH = 8.0) for 1 min,
- 7. 0.1 M TEA- 0.25% acetic anhydride for 10 min,
- 8. PB for 1 min,
- 9. 70, 80, and 90% ethanol for 15 s each, and.
- 10. 100% ethanol for two 15-s intervals.

The gene transcripts for *mzpc5* were visualized by hybridization with digoxigenin (Dig)-labeled probes of the amplified cDNA (485 base pairs; bp) following RT-PCR using primer set 1 (Fig. 2B) and a commercial kit (DIG DNA Labeling and Detection kit, Roche Diagnostic Co. Indianapolis, IN). The expression signals were observed with an Olympus BH-2 microscope at the CAMI Core facility in the UC Davis Center for Health and the Environment.

### Antibody production

The Immune Epitope Database and Analysis Resource [40] was searched to identify potential mZPC5 peptide sequences that could be used as target antigens in producing anti-mZPC5 antibodies. Only one peptide sequence, “SGALAAHELPPVLHKIHKT”, was identified; no similar sequence existed for liver-expressed Chgs. or ovary-expressed ZPs. The mZPC5 peptide antigens and anti-mZPC5 antibodies were produced by Sigma Aldrich (Tokyo, Japan).

### Preparation of tissue extracts from wild-type and *chg.l-/-* fish and chorion lysates from wild-type ovarian oocytes for sodium-dodecyl-sulfate polyacrylamide gel electrophoresis

Tissue-extract samples were collected to determine whether mZPC5 was present in the livers and ovaries of the mature *chg.l* + */* + (normal wild-type) and *chg.l -/-* females. Chorion-lysate samples were obtained only from the *chg.l* + / + (normal wild-type) female medakas. The tissue-extract and chorion-lysate samples were prepared following the procedures described by Murata et al. [12–14]. First, after bleeding the fish as described above, each liver and ovary was dissected, transferred into a 1.5-ml tube containing 100 µl of TBSE (Tris-buffered saline (TBS) containing 40 mM EDTA), and homogenized. After the homogenized tissue was centrifuged at 14,000 rpm for 10 min at 4 °C, the supernatant was diluted at a 1:1 volumetric ratio with sodium-dodecyl-sulfate (SDS) sample buffer containing 2-mercaptoethanol. The diluted supernatant was then boiled and used as the tissue sample for SDS–polyacrylamide gel electrophoresis (PAGE) analysis. The supernatant was boiled for 5 min and stored at –20 °C for future use.

The inner layer of the oocyte envelope (i.e., chorion lysate) of each wild-type ovarian oocyte was prepared using procedures described by Murata et al. [12]. Briefly, after bleeding the female as previously described, ovulated oocytes were isolated by cutting the abdominal cavity. The ovaries were dissected from the fish and transferred into a dish containing ice-cold TBSE. Then, the germinal epithelium was avulsed with fine forceps and scissors to release the ovarian oocytes from each ovary. The cytoplasmic contents of the oocyte (ca. > 600 µm) were removed by making a small nick with ophthalmic scissors, suctioning with a thin, smooth-tipped glass pipet to avoid damaging the membrane, and flushing with ice-cold TBSE. Each isolated (ooplasm-free) chorion was transferred into a petri dish containing excess fresh ice-cold TBSE and rinsed by pipetting additional fresh ice-cold TBSE onto it. All chorions were transferred to one 1.5-ml tube with the ice-cold TBSE rinse and centrifuged at 14,000 rpm for 20 min. Afterward, the supernatant was replaced with fresh ice-cold TBSE.

The prepared chorions were minced using surgical eye scissors and centrifuged at 14,000 rpm for 20 min. After centrifugation, the supernatant was removed from the sample and added to 100 µl of 0.05 N sodium hydroxide (NaOH). The precipitate was rinsed by pipetting in this alkaline solution. Immediately afterward, the 0.05 N NaOH was replaced with 100 µl of 0.05 N NaOH solution containing 0.05 M sodium chloride (NaCl), and the resulting sample was homogenized and incubated at 60 °C for 30 min. After incubation, the samples were centrifuged at 14,000 rpm for 20 min. The supernatant was mixed at a volumetric ratio of 1:1 with 2 × SDS-sample buffer containing 2-mercaptoethanol, boiled for 5 min, and stored at –20 °C for later use in SDS-PAGE and/or Western blotting assays.

### SDS-PAGE and Western blot assays on the tissue-extract and chorion-lysate samples

After thawing for 30 min at room temperature, the tissue-extract and chorion-lysate samples were centrifuged at 14,000 rpm. The supernatant was used for SDS-PAGE and Western blot analysis.

### SDS-PAGE analysis

The proteins in the SDS-PAGE samples were separated by mass using SDS-PAGE gels, according to Laemmli [41]. A 10% SDS-PAGE gel was used for the liver extracts, and an 8% SDS-PAGE gel was used for the ovary extracts. The immunoreactive proteins were detected following western blotting analysis.

### Western blot analysis

Western blot analysis was performed following the methods of Murata et al. [12–14] using anti-mZPC5, anti-Chg.L, and anti-Chg.H primary antibodies, as well as horseradish peroxidase (HRP)-conjugated goat anti-rabbit immunoglobulin (Ig)G (A16096; Invitrogen, Waltham MA USA) and HRP-conjugated rabbit anti-mouse IgG (62–6520: Thermo Fisher Scientific, Waltham, MA USA) secondary antibodies. The specificity of the anti-Chg.L and anti-Chg.H antibodies was demonstrated previously [32]. All primary and secondary antibodies were diluted at volumetric ratios of 1:2000 and 1:5000, respectively, using 2% bovine serum albumin (BSA)-TBS containing 0.05% Tween-20 and 2% pre-immune goat serum (BTTBSG) as the diluent.

After the SDS-PAGE was performed, the proteins in the gel were transferred onto a polyvinylidene fluoride **(**PVDF) membrane (Immobilon-P; Millipore Co. Billerica, MA, USA). The proteins on the membrane were then stained with Coomassie Brilliant Blue R-250 (CBB: Thermo Fisher Scientific Waltham, MA USA) to determine their molecular masses.

After treatment of proteins on the membrane with the primary and secondary antibodies, the immunoreactive protein bands were visualized using a 3,3′,5,5′-tetramethylbenzidine (TMB) substrate kit (Vector Lab. Inc. Burlingame. CA).

### Mass spectrometry analysis of immunoreactive proteins

A sterile razor blade was used to manually excise the 72–74-kDa protein bands that reacted with anti-mZPC5 antibodies in the ovarian-tissue and chorion-lysate extracts. The extracts were then minced and placed into individual tubes for mass spectrometry analysis to identify the immunoreactive proteins. After in-gel digestion, the peptide sequences were identified via tandem mass spectrometry (MS/MS) analysis. Computational analysis of the output results was performed in the Proteomics Core Facility at the UC Davis Genome Center, following their procedures [42].

### Double-staining with anti-mZPC5 and anti-Chg.H antibodies for immunohistochemical analysis

The procedures used to prepare tissue sections for immunohistochemistry were the same as those described in the “In situ hybridization” section above. All primary and secondary antibodies used for immunohistochemical staining were diluted at a volumetric ratio of 1:1000 using BTTBSG. Over 4 days, the tissue sections underwent the same basic treatment of overnight incubation at 4 °C with an antibody and rinsing with TBS before the next incubation period. Anti-mZPC5 primary antibody, Alexa Fluor® 488 goat anti-rabbit IgG secondary antibody (Invitrogen, Carlsbad, CA 92,008 USA), mouse anti-Chg.H primary antibody, and Alexa Fluor® 568 goat anti-mouse IgG secondary antibody (Invitrogen, Waltham MA USA) were used on days 1–4, respectively. The Alexa Fluor® 488 and 568 secondary antibodies were used to visualize tissues immunoreactive to the anti-mZPC5 and anti-Chg.H primary antibodies, respectively, and to determine whether the mZPC5 and Chg.H proteins were co-localized in the same tissues. After the final rinse with TBS, each glass slide-mounted tissue section was covered with mounting medium (i.e., 90% glycerol, 10% 50 mM Tris–HCl, pH = 7.4, containing 0.15 M NaCl (TBS) with 50 mM N-propyl gallate) and a glass coverslip for microscopic analysis.

It should be noted that selected sections were incubated with diluent alone (i.e., BTTBSG; no antibodies), following the same general procedural sequence as described in the previous paragraph to serve as negative controls. The negative controls were used to ensure that the positive signals in the sections came from specific reactions with the primary antibodies, not the diluent.

### Immunofluorescence imaging of the ovary sections

The double-stained (i.e., primary and secondary antibody-labeled) ovary sections were observed using an Olympus Fluoview 500 confocal laser scanning microscope mounted onto an Olympus BX61 upright, fixed-stage microscope (Olympus Imaging America Inc., Center Valley, PA), each equipped with fluorescence water immersion objectives.

## Results

### Evidence of *mzpc5* expression in the ovary, but not in the liver, of spawning female medakas

Figure 2A shows the predicted genomic structure of *mzpc5* and the primer positions used to 1) identify *mzpc5* expression in the liver and ovary of spawning females, and 2) clone cDNA after RT-PCR. *Mzpc5* is located on Chromosome 8, and its total length is 1.79 kilobase pairs (kbp), spanning 10 exons (blue squares, Fig. 2A) and 9 introns. Expression of *mzpc5* was only detected in the RNA obtained from spawning female ovaries and not from the livers of spawning female or mature male livers (Fig. 2B) using specific primer set 1 (F3 and R3 in Fig. 2C). Figure 2C shows the nucleotide and predicted amino acid sequences of the *mzpc5* cDNA newly cloned in this study. The cDNA consisted of 1539 nucleotides encoding the full-length predicted amino acid sequence of mZPC5 (498 amino acids). The predicted signal sequence cleavage site was located between ^25^Alanine (A) and ^26^Phenylalanine (F) according to PSORT [37], a computer program for the prediction of protein localization sites in cells. The similarities between the nucleotide sequence of the *mzpc5* cDNA and the corresponding predicted amino acid sequence and those of the *zpc5* cloned by Kanamori (cDNA accession number AAN31192) were 99.1% and 99.4%, respectively. The portions of the nucleotide and predicted amino acid sequences that are different in our *mzpc5* versus those of Kanamori are shown with bold blue letters (^312^Glycine and ^343^Lysine) in Fig. 2C. Based on our analyses, in the predicted amino acid sequence encoded by *mzpc5*, there were 17 amino acids possibly linked to O-linked oligosaccharides and two peptides linked to N-linked oligosaccharides after port-translational modification in the ovary.

The localization of *mzpc5* expression in the ovary was examined by performing in situ hybridization with Dig-labelled specific RNA as the probe.

### The expression of *mZPC5* in the ovary

Sense (control) and anti-sense probes for the 485-bp, Dig-labeled RNA were prepared using *mZPC5*F3 and *mZPC5*R3 primers. No signals were observed in the ovary using a Dig-labeled sense probe.

**Fig. 3:**
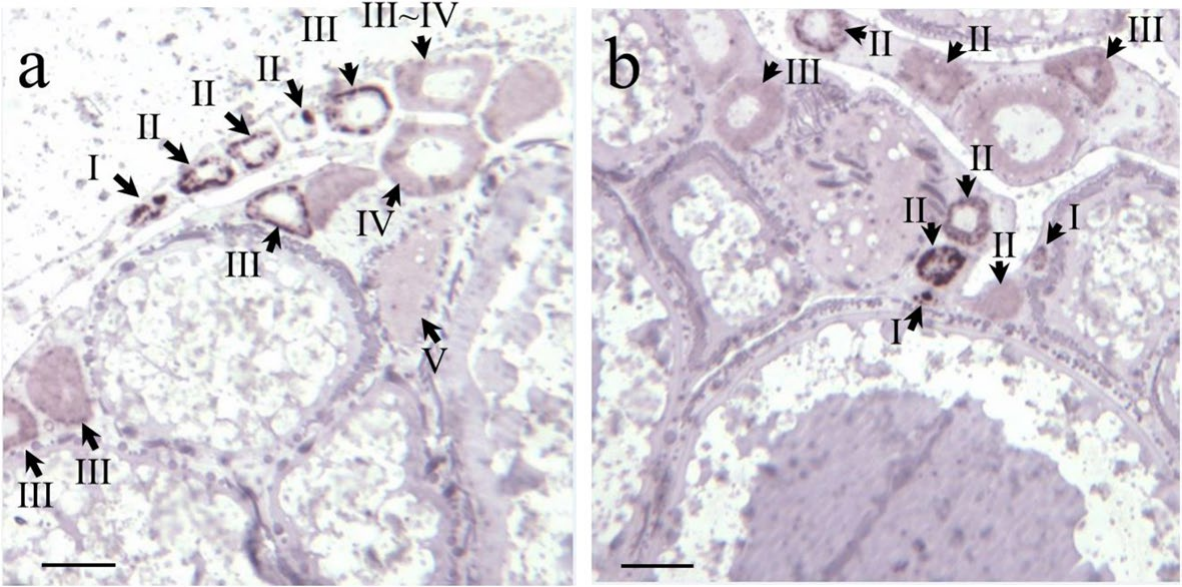
The expression of *mzpc5* in the ovary. Brightfield micrographs of *mzpc5* transcripts detected in the ovary of a spawning female medaka using in situ hybridization. Scale bar = 100 µm. The arrows point to *mzpc5* expressed in oocytes of varying sizes. Panels a and b show different portions of the same ovary. Only small oocytes (stages I–III) showed intense signals of *mzpc5* expression

The in situ hybridization analysis revealed intense signals (dark staining) in the previtellogenic (stage I–III) oocytes (Fig. 3, black arrows). As the size of the oocytes increased, the signal weakened. Finally, signals were not detected in the mature oocytes (not shown).

### Anti-mZPC5 antibody immunoblotting analysis of chorion lysates and ovary and liver extracts obtained from spawning female medakas

**Fig. 4:**
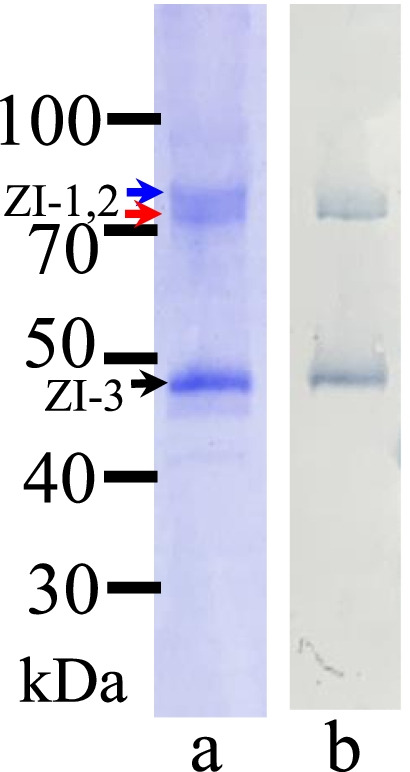
Immunoreactivity of the anti-mZPC5 antibody and detection of mZPC5 in the chorion lysates. **a**: CBB-stained proteins in the chorion lysate, **b**: proteins immunoreactive to anti-mZPC5 antibody. The numbers (30–100) on the y-axis represent the molecular masses of the detected proteins in kilodaltons (kDa). The top (blue), middle (red), and bottom (black) arrows represent the positions (molecular masses) of ZI-1, ZI-2, and ZI-3, respectively. ZI-1 and -2 fall between 100 and 70 kDa, and ZI-3 falls between 50 and 40 kDa on the y-axis

As shown in Fig. 4a (blue/top, red/middle, and black/bottom arrows, respectively), medaka chorions are composed of three major proteins, ZI-1 (76 kDa), ZI-2 (74 kDa), and ZI-3 (49 kDa). Liver-expressed Chg.H and Chg.Hm accumulate in the chorion as ZI-1 and -2, and Chg.L accumulates as ZI-3 after their chemical modification (conformation change) during chorion formation [18]. Figure 4b shows the immunoreactive proteins in the chorion lysate detected by the anti-mZPC5 antibodies. The two proteins appeared at almost the same positions as ZI-1/-2 and ZI-3.

**Fig. 5:**
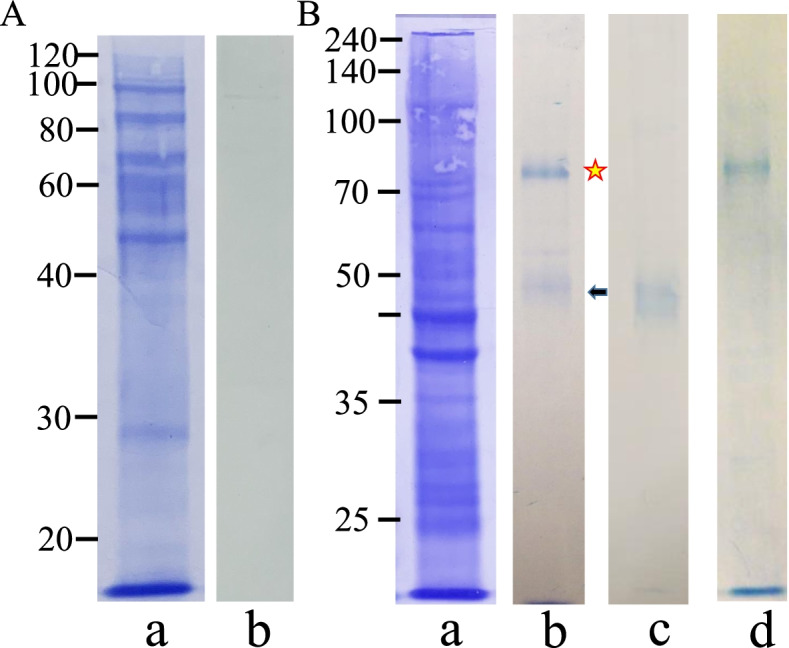
Specificity of the mZPC5 antibody and detection of mZPC5 in female tissue extracts. SDS-PAGE results are shown for liver (A) and ovary (B) tissue extracts of spawning females. **A:** the 10% SDS-PAGE pattern of CBB-stained proteins in the female liver extracts (Aa) and the associated proteins that were immunoreactive to the anti-mZPC5 antibody (Ab). **B:** the 8% SDS-PAGE pattern of CBB-stained proteins in the female ovary extracts (Ba) and the associated proteins that were immunoreactive to the anti-mZPC5 (Bb), anti-Chg.L (Bc), and anti-Chg.H (Bd) antibodies, respectively. Symbols – yellow star: ~ 74-kDa proteins reactive to the anti-mZPC5 anyibody, black arrow: ~ 50-kDa proteins reactive to the anti-mZPC5 antibody

As shown in Figs. 1 and 4, the expression of *mzpc5* was only detected in the sample obtained from spawning female ovaries, and its gene product, mZPC5, was present in the chorion lysate. Figure 5 shows the proteins that were immunoreactive to the anti-mZPC antibodies in the tissue extracts obtained from the wild-type spawning females. No immunoreactive proteins were detected in the males (data not shown) or in female liver tissue extracts treated with the anti-mZPC5 antibodies (Fig. 5Ab).

Two immunoreactive proteins were detected in the ovary extracts using anti-mZPC5 antibodies (Fig. 5Bb). Their associated SDS-PAGE patterns appeared similar to that in the chorion lysate (Fig. 4b). However, the immunoreactivity of the proteins at the 49-kDa position (i.e., the expected location of Chg.L) appeared weaker (black arrow in Fig. 5Bb versus 4b). Relatively stronger signals (yellow stars in Figs. 5Bb and Bd) were detected in the region corresponding to Chg.H and Chg.Hm (MW = 74–76 kDa). However, the size of the immunoreactive protein detected with the anti-mZPC5 antibodies was somewhat smaller than that detected with anti-Chg.H antibodies in the ovary extracts. These data strongly suggested that mZPC5 exists in the ovary but not in the liver.

### MS/MS analysis of proteins immunoreactive to the anti-mZPC5 antibodies

Based on the predicted amino acid sequence, the MW of the coding region of mZPC5 is 52.6 kDa (Fig. 2C). However, the MW of the immunoreactive protein detected in ovary extracts using anti-mZPC5 antibodies (Fig. 5Bb) was 74-kDa. Thus, peptide sequencing of the 74-kDa protein was performed via MS/MS to identify whether it was mZPC5 or a related protein. The 74-kDa protein is highlighted in Fig. 5Bb with a star.

One hundred eighty-five peptides originating from 22 different proteins, including vitellogenin, collagen, medaka keratin, Chg.H, Chg.Hm, and mZPC5, were detected in ovarian-tissue extracts by MS/MS analysis. One hundred thirty-six peptides originating from 9 different proteins, including vitellogenin, actin, Chg.H, Chg. Hm, and mZPC5, were also detected by MS/MS analysis in chorion lysate extracts. The peptide sequences for mZPC5, “ALSIPGVFNPR (344–354)” and “SGLNTIEK (420–427),” located in the predicted amino acid sequence in Fig. 2C, were detected. (The numbers in parentheses indicate the positions in the amino acid sequence in Fig. 2C). These peptides do not exist in Chg.L; therefore, we determined that the immunoreactive protein with a MW of 74–76 kDa includes mZPC5 or the antigen peptide for the anti-mZPC5 antibody.

### Immunohistochemical localization of the mZPC5 protein in the ovaries of mature females

**Fig. 6:**
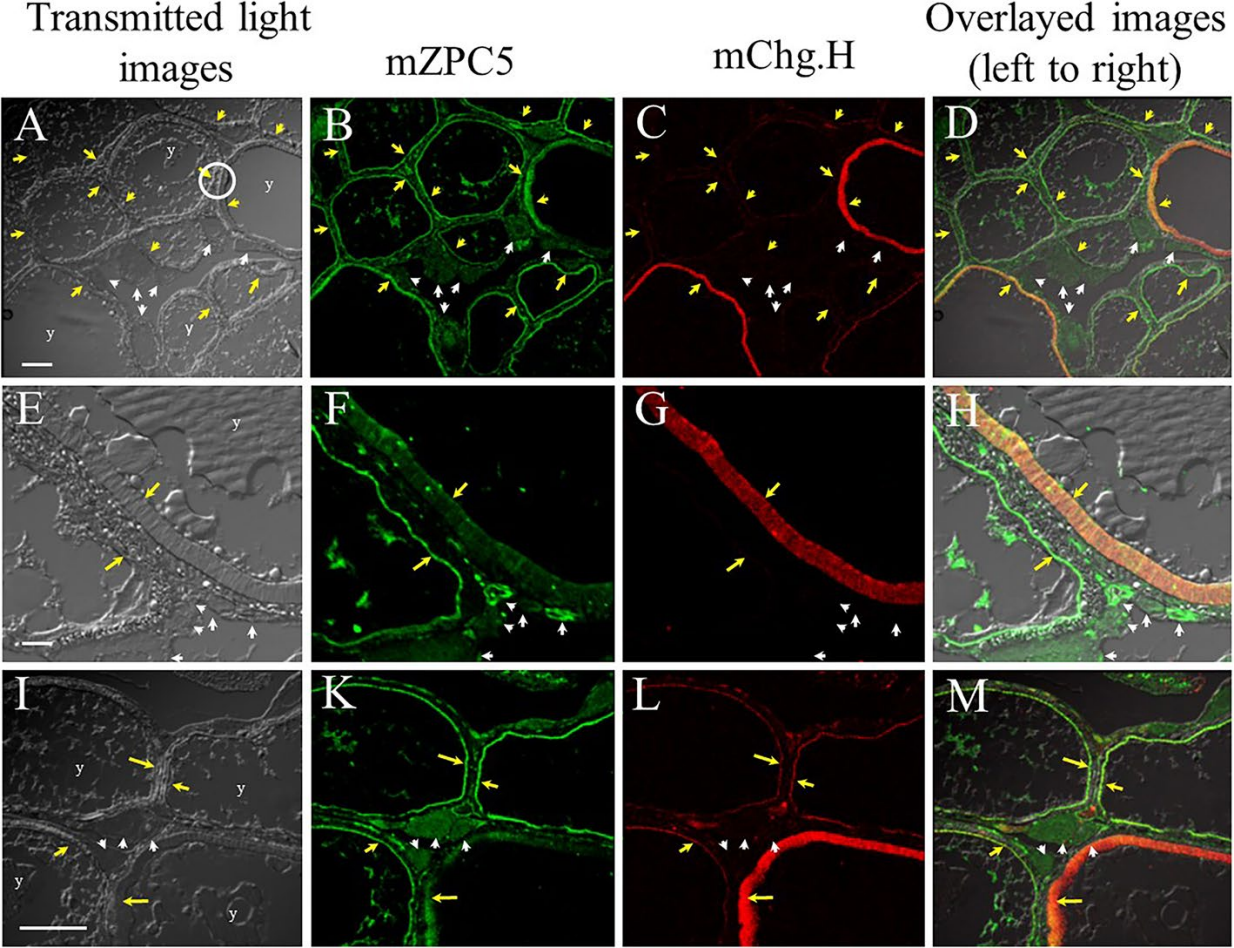
Immunohistochemical detection of mZPC5 in the ovaries using anti-mZPC5 and anti-Chg.H antibody. All images in the same row show the same tissue section. The first three columns (left to right) show the tissues under different microscope light settings, while the last column shows images from columns one, two and three overlaid. **A, E, and I:** transmitted light images of oocytes in the ovaries of sexually mature females. **B, F, and K:** the portions of the tissues reacted with the anti-mZPC5 antibody (green). **C, G, and L:** the parts of the tissues reacted with the anti-Chg.H (positive control) antibody (red) in the same ovarian oocyte as A, E, and I, respectively. The white-circled area in Fig. 6A was magnified 2.5 times in Fig. 6E–H to visualize structural details in the developing chorion and ovarian oocyte. Symbols – y: yolk, yellow arrows: the position of the chorion, white arrows: early developed oocytes. Scale bars in A, E, and I represent 100, 40, and 100 µm, respectively. The developing stage of oocytes was determined following guidance from Iwamatsu et al., 1988 [34]

The western blotting and MS/MS analyses revealed that mZPC5 was present in the ovary tissue extracts, but not in the liver tissue extracts, from mature female fish (Fig. 5). Thus, immunohistochemical observations of the spawning female ovary sections were performed. Anti-mZPC5 and anti-Chg.H antibodies were used in conjunction with confocal microscopy. The anti-Chg.H antibody served as a positive control to locate the chorion in each ovarian oocyte and immunoreactive proteins in the ovary (Fig. 6).

The primary antibodies were omitted in the negative control, and no signals were detected (data not shown). As shown in Fig. 6, intense green fluorescence signals representing mZPC5 were detected in the chorion of early-stage (i.e., stage III–V; yellow arrows in Fig. 6B, F, and K) oocytes. The ooplasm of stage I–III oocytes was also stained with anti-mZPC5 antibody (white arrows in Fig. 6B, F, and K) but lacked the characteristic red staining from the anti-Chg.H antibody (Fig. 6C, G, and L). The green signals were more intense in the small than in the large oocytes, supporting the observation that the mZPC5 signal weakens as the oocytes increase in size. Figure 6D, H, and M clearly show that mZPC5 and Chg.H co-localize within the chorions of the larger oocytes. These results are also supported by our in vitro hybridization experiments (Fig. 3). The decreased *mzpc5* expression in growing oocytes weakens the immunohistochemical detection signals of mZPC5 in the ooplasm. However, the timing of the appearance of mZPC5 and Chg.H in the chorions is different. While Fig. 6B, F, and K clearly show that mZPC5 is present in the chorions of small oocytes, the Chg.H signals were very weak (Fig. 6C, G, and L) in those same oocytes. As the oocytes grow and the chorions develop just outside of the oocyte plasma membrane, the Chg.H signal strength increases, illustrating an opposite temporal pattern from that of the mZPC5 signal.

### Western blot and immunohistochemical analyses of female *chg.l*-KO tissues and oocytes

**Fig. 7:**
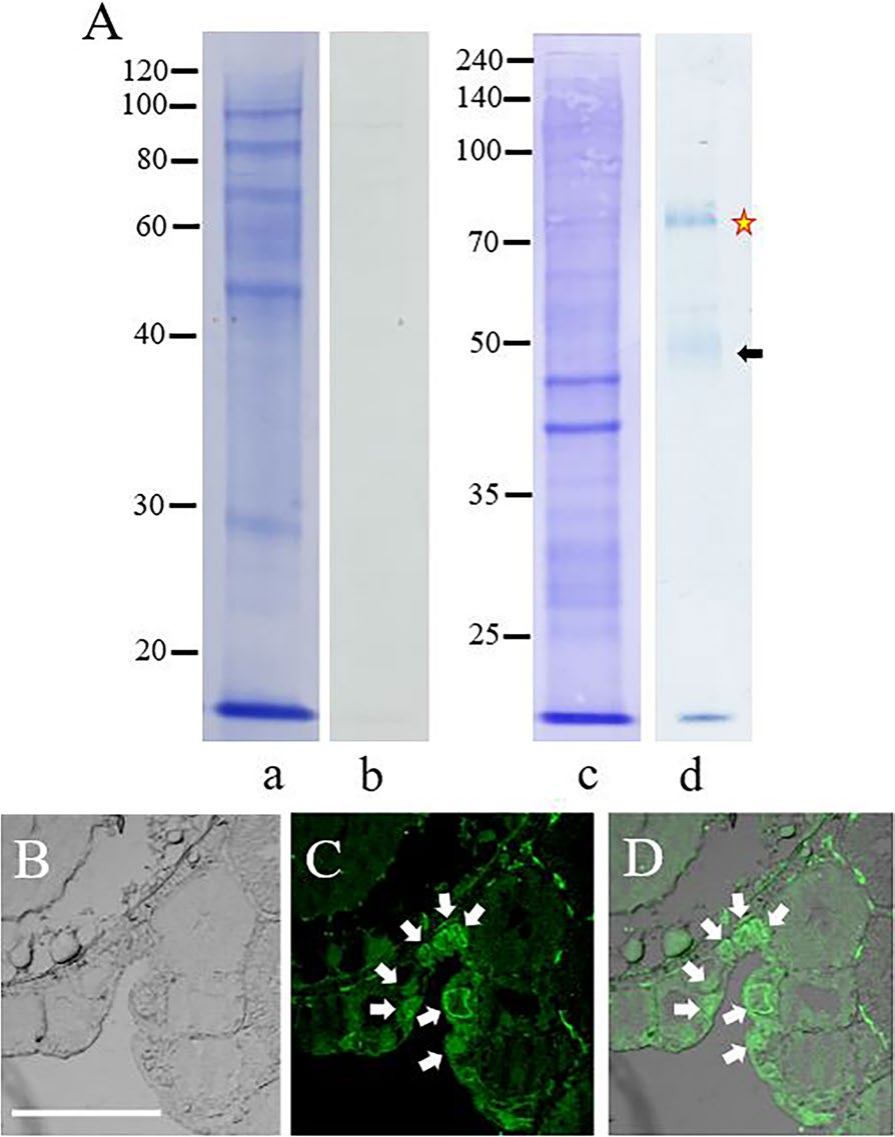
Detection and localization of mZPC5 proteins in *chg.l* -/- females. **A**: SDS-PAGE and western blot assays of the liver (7Aa and 7Ab) and ovary extracts (7Ac and 7Ad) using anti-mZPC5 antibody. 7Aa: the 10% SDS-PAGE pattern of proteins in the liver extracts stained with CBB. Ab: proteins immunoreactive to the anti-mZPC5 antibody in the liver extracts. Ac: 8% SDS-PAGE pattern of proteins in the ovary extract stained with CBB. Ad: proteins immunoreactive to the anti-mZPC5 antibodies in the ovary extracts. **B**–**D**: confocal microscope observations of the same ovarian oocytes (ovarian stage less than V) to identify the locations of immunoreactive proteins in the ovarian oocytes using anti-mZPC5 antibodies as probes. **B:** a transmitted light image provided to illustrate the exact morphology of the ovarian oocytes without immunochemical staining. **C:** same as B but including the localized anti-mZPC5-immunoreactive proteins. **D**: overlaid images B and C. Symbols: yellow star and black arrow in Ad – immunoreactive proteins with 72–72 kDa and 50 kDa molecular masses, respectively; white arrows in C and D – oocytes that were immunoreactive to anti-mZPC5 antibodies. Scale bar in Panel B = 100 µm

The CBB-stained liver-extract proteins of the mature *chg.l -/-* (Fig. 7Aa) and wild-type (Fig. 5Aa) females were similar. No detectable liver-extract proteins were immunoreactive to the anti-mZPC5 antibody in the *chg.l -/-* females. The results in Fig. 7Ab strongly suggest that *mzpc5* is not expressed and mZPC5 is not produced in the livers of spawning normal or *chg.l -/-* females.

The anti-mZPC5-reactive proteins (Fig. 7Ad) appeared in the same locations as those found in wild-type ovary extracts (Fig. 5Bb) and chorion lysates (Fig. 4b). An intense signal was obtained for the protein in the approximate position of Chg.H (Fig. 7Ad, star) compared to the signal for the protein in the approximate position of Chg. L (Fig. 7Ad, black arrow).

As shown in Fig. 7B, C, and D, stage I-III ovarian oocytes in the *chg.l -/-* female ovaries were immunoreactive to the anti-mZPC5 antibodies in the ooplasm and the portion of the oocytes just outside of the ooplasm (white arrows in Fig. 7C and D). This finding was similar to that for normal oocytes at the same developmental stages (Fig. 6F and H).

### Immunohistochemical detection of mZPC5 in the ovarian oocytes of *chg.l -/-* females

**Fig. 8:**
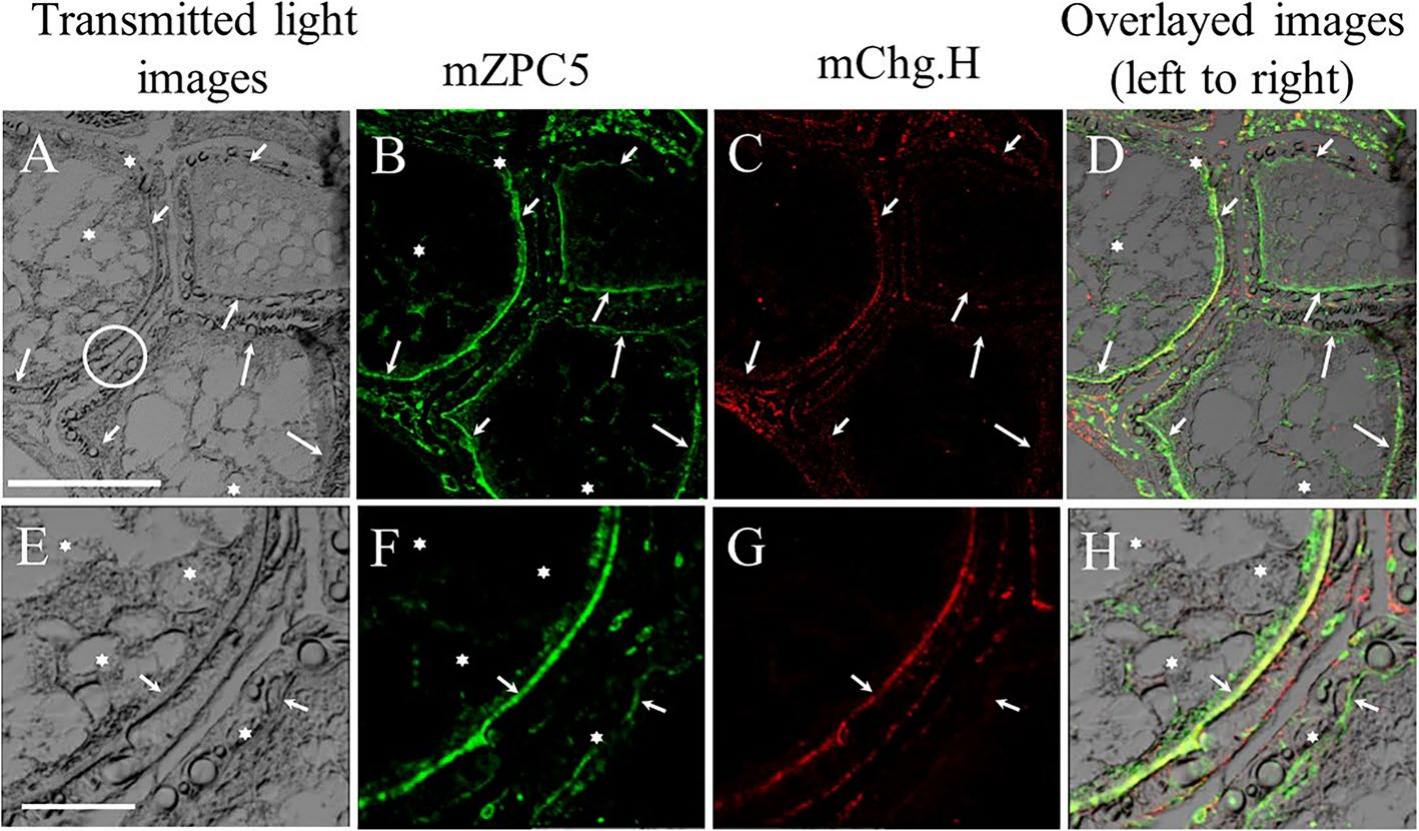
Immunohistochemical detection of mZPC5 in a *chg.l-/-* female ovary. **8A** and **8E**: Transmitted light image of an ovarian oocyte in the ovary of a sexually mature female. **8B** and **8F**: The same ovarian oocytes shown in 8A and E, respectively, but with highlighted issue regions immunoreactive to anti-mZPC5 antibodies. **8C** and **8G**: The same ovarian oocytes shown in **8A** and **8E**, respectively, but with highlighted issue regions immunoreactive to anti-Chg.H antibodies. **8D**: Transmitted light image 8A overlaid with **8B** and **8C**. **8H**: Transmitted light image **8E** overlaid with **8F** and **8G**. White arrows: position of the chorion. It should be noted that **8E** to **8H** show magnifications of the white-circled area in **8A**. Symbols – white stars: the location of ZPC5 in the ooplasm. Scale bars in **8A** and **8E** represent 100 and 20 µm, respectively. The developing stage of the oocytes was determined following Iwamatsu et al. 1988 [34]

Figure 8 shows the localization of anti-mZPC5-immunoreactive proteins in *chg.l -/-* ovarian oocytes at approximately stage V of development (Fig. 8B and F). Intense signals were detected in the chorions of developing oocytes, and weak signals were detected in the ooplasm, especially below the chorion, using anti-mZPC5 antibodies as probes (white stars in Fig. 8B, D, F, and H). Less immunoreactivity to anti-Chg.H antibody was observed in the *chg.l -/-* (Fig. 8C) versus *chg.l* + */* + (Fig. 6G) ovarian oocytes. Chg.H was localized in the *chg.l -/-* ovarian oocytes—as shown in the white-circled area in Fig. 8A (magnified in Fig. 8E–H)—and in their thin chorions (Fig. 8G).

## Discussion

Among the oviparous members of teleost fish, the chorion glycoproteins are synthesized in the liver and/or developing oocytes in the ovaries of spawning females. The liver-expressed chorion glycoproteins are Chgs., and oocyte-expressed proteins containing a ZP domain are called ZP proteins. The expression of Chgs. in the spawning female liver is induced by estrogen [14, 15, 17, 19], and the expression of oocyte-specific ZP proteins is thought to be controlled by the transcription factor FIGα, as in mammals [28, 30, 43]. In medakas, Chg.L is the major component of the chorion that corresponds to ZPC in mammals [19, 44]. In 2022, a *chg.l-/-* medaka line was established [32]. The *chg.l-/-* females produced oocytes with very thin chorions and spawned string-like material containing infertile “smashed eggs.” Overall, the *chg.l-/-* chorion was so thin that it was not strong enough to support and maintain the oocyte’s structural shape and integrity, which usually allow it to withstand the pressures exerted against the chorion during spawning events. Along with the thin chorion, a very thin and fragile matrix formed and surrounded the oocytes [32].

Of interest, *zpc*^−/−^ female mice produce ZP-free oocytes without forming a proper cumulus-oocyte complex [45]. Chg.L in medaka is homologous to ZPC in mammals. However, during evolution, the molecules comprising the extracellular matrix of the chorion also evolved such that each species acquired its own fertilization mechanisms. One of the apparent differences in the chorion structures of fish and mammals is that the eggs of most bony fish (Osteichthyes, Euteleostomi) have unique structures called micropyles on the surface of the chorion that permit only a single sperm to penetrate the oocyte [46].

In 2000, Kanamori [29] confirmed the expression and genomic structures of ZP-proteins in the ovary [30]. However, the functions of these proteins remain undetermined, specifically regarding whether they function as components of the chorion. To determine this, we first analyzed the expression and localization patterns of ZP-proteins in the ovary and biochemically characterized one of the ZP-domain-containing proteins, mZPC5, by performing in situ hybridization and biochemical and immunohistochemical analyses using specific antibodies. In the wild-type medakas, *mzpc5* expression was only detected in the spawning females’ ovaries, not in the livers of spawning females (Fig. 2B) or males (Fig. 1Bc). In the ovaries, intense signals were obtained in the previtellogenic (stage I–III) oocytes following in situ hybridization analysis. As oocytes grew larger, the signals became weaker (Fig. 3a and b). The synthesis of the chorion glycoproteins in cyprinids (goldfish, carp, and zebrafish) appears to be restricted to the oocyte [25–28]. The strength of the *mzpc5* expression detected by in situ hybridization (Fig. 3) may depend upon the hybridization conditions used in the present study. It is also possible that *mzpc5* expression is not restricted to small (stage I–III) oocytes. Rather, it may be expressed ubiquitously but weakly in the ooplasm of all growing oocytes during oogenesis. However, our immunohistochemical results suggested that mZPC5 production decreased in the ooplasm of growing oocytes and accumulated in a portion of the chorion (Figs. 6, 7, and 8). Considering all of our findings, we think that *mzpc5* is strongly expressed only in small (stage I–III) oocytes, and the resulting mZPC5 may induce (initiate) the chorion formation mechanism in the growing oocytes. Additional research is needed to elucidate the functions of mZPC5 in choriogenesis.

In carp and zebrafish, *zp* expression has been observed in the ooplasm of previtellogenic oocytes; *zp* expression has not been observed in vitellogenic oocytes or other ovarian cells [25–28]. The expression pattern of *mzpc5* is similar to that of *zp* in carp and zebrafish. Thus, our results may also suggest that the expression of *mzpc5* in medakas may be controlled in a manner specific to the oocyte’s developmental stage. In mice, the FIGα helix-loop-helix transcription factor was identified as regulating the expression of *zp* genes in the ovary [43]. In medakas, the expression patterns of the FIGα transcription factor [30] and *mzpc5* (Fig. 3) are quite similar, suggesting that the expression of *mzpc5* is possibly regulated by FIGα, as are mammalian ZP proteins. However, in medakas, the mechanism of *mzpc5* expression may be more complicated. For example, the medaka testis-ova condition (i.e., the occurrence of oocytes in the testis of male fish) is marked by enhanced *mzpc5* expression enhanced by the male fish’s exposure to 17 alpha-ethinylestradiol (EE2), and *mzpc5* is more sensitive to EE2 than m*zpc1-4* are [31]. Future work should determine how FIGα and EE2 are involved in the molecular mechanisms of *mzpc5* expression during oogenesis.

Based on our western blotting analysis (Fig. 4B), the anti-mZPC antibodies bound to a protein with a MW (74–76 kDa) that was higher than that calculated (52.6 kDa) from the predicted amino acid sequence in the coding region of *mzpc5* (Fig. 2C). MS/MS sequencing of the immunoreactive protein revealed the presence of mZPC5 peptides (Fig. 2C). Therefore, we identified that 74- to 76-kDa protein as a modified mZPC5 form or a modified protein complex that includes specific antigen peptides in the mZPC5 protein. In the predicted amino acid sequence, there were 17 amino acids possibly connected to O-linked oligosaccharides (blue-circled amino acids in Fig. 2C) and two peptides connected to N-linked oligosaccharides (blue-boxed peptides in Fig. 2C). Because of the oligosaccharide chains, the MW of the mZPC5 protein may be greater than that predicted by the predicted amino acid sequence.

Additionally, when we isolated the chorions, we could not completely remove the cytosolic contents of the oocytes. If the mZPC5 molecule undergoes modifications and accumulates in the developing chorion during chorion formation, it should be possible to detect proteins of different MWs that bind to anti-mZPC5 antibodies. Interestingly, we detected two peptides, “ALSIPGVFNPR (344–354)” and “SGLNTIEK (420–427), by MS/MS analysis (Fig. 2C). These proteins were located near the C-terminus of mZPC5. This result suggests that during the accumulation of mZPC5 at the chorion, the peptide containing the C-terminal region of mZP5 may play an important role, along with other components (e.g., Chgs.), in the construction of the chorion.

While the functional role of mZPC5 was not examined mechanistically in the present study, it was localized in the ovaries, as shown in Fig. 6. The ooplasm of previtellogenic oocytes was stained by the anti-mZPC5 antibody (white arrows in Fig. 6B, D, K, and M). Intense signals were detected in the oocyte at the rudimentary chorion stage (stage III) and early vitellogenic stage (stage V). However, as the oocytes enlarged and the chorions became thicker, the mZPC5 signals in the chorions became weaker. In contrast, the signal representing Chg.H became increasingly stronger. These results suggested that the weakening mZPC5 signals were consequences of the increased thickness of the chorions in the growing oocyte. The subsequent dilution of the mZPC signals was likely due to the consumption of expressed *mzpc5*. Our results indicated that *mzpc5* was expressed intensely in small (stage I–III) oocytes, with expression decreasing and/or stopping with oocyte growth (Fig. 3).

**Fig. 9:**
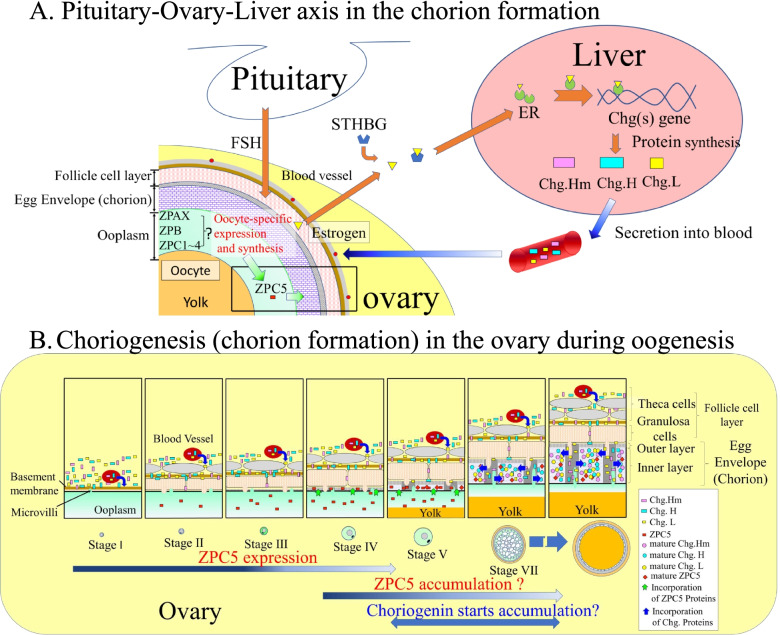
Summary of chorion formation in medakas. Choriogenin (Chg.) expression is induced by estrogen in the liver. Chgs. are secreted into the blood (A: gradient blue arrows) and transferred to the ovary (A), where they accumulate in the chorion by way of the pore canals (B: solid dark blue arrows) after modification. Oocyte-expressed ZPC5 (A) and possibly other ZPs pass through the oocyte plasma membrane (B: green arrows) and accumulate in the chorion. It is still unknown if ZPC5 accumulates in the chorion through the pore canals

Accumulation of the Chgs. and mZPC5 proteins may occur at different times during chorion formation. In medakas, mZPC5 may be secreted from the ooplasm through the oocyte plasma membrane into the space where the chorion architecture develops, thus establishing a foundation for chorion thickening with oocyte growth (Fig. 9, green arrows). Furthermore, as shown in Figs. 7 and 8, mZPC5 is present in the ovarian oocytes of *chg.l-/-* females that produce oocytes with very thin, soft chorions. These results suggest that during chorion formation, mZPC5 may interact with other chorion glycoproteins such as Chg.H, Chg.Hm, and other ovary-expressed ZP domain-containing proteins. However, the interaction of mZPC5 with Chg.H, Chg.Hm, and other ovary-specific chorion proteins may be quite weak or insufficient to form a chorion of normal thickness and compensate for the loss of function of Chg.L [32].

A previous study [45, 47] showed that homozygous mutant *zpc*-/- mice had germinal-vesicle-intact, zona-free follicles, while other protein components (ZPA and ZPB) were detected at the surface of the zona-free oocytes. In the present study, we observed similar phenomena. The chorions in the mature oocytes produced by *chg.-/-* females were very thin and weak. Thus, they were not strong enough to support the spherical structure of oocytes during the ovulation and spawning processes, resulting in spawned smashed eggs [32]*.* However, oocyte-specific mZPC5 is secreted from the ooplasm of the previtellogenic oocyte and deposited on the outer surface of the oocyte plasma membrane, creating the thin layer of the chorion just exterior to the plasma membrane (Fig. 7B–D). It is also secreted slightly later, initiating the accumulation of Chgs. from the liver and potentially interactions with all egg-envelope-related proteins to create the 3D structure of the mature chorion. Previously, we observed a similar phenomenon in white sturgeon, *Acipenser transmontanus,* oocytes [48]. In white sturgeon, the chorion glycoproteins are synthesized in the liver of mature females, the oocytes, and possibly the follicle cells of the ovary. In sturgeon oogenesis, the chorion first develops as a single layer and differentiates into two layers as the oocyte develops from the oocyte-side toward the follicle cell layer [48]. The phylogenetic distance between sturgeon and medakas is extreme; however, the molecular mechanisms of chorion formation may have been conserved during evolution. Similar phenomena have been reported in the euteleostean gilthead seabream, *Sparus aurata*. In the euteleostean gilthead seabream, besides the liver-expressed Chgs., the homolog of the ovary-expressed ZPX gene was identified as a component of the inner layer of the chorion [49, 50]. Currently, in medakas, it remains unknown if mZPC5 is transported from the ooplasm through the pore canal of the chorion as Chg. proteins are [16].

In 2008, using purified trout and mouse ZPCs separately, Darie et al. [51] and Litscher et al. [52] showed that ZP-domains assemble each other to form a higher-order architecture in the chorion. Choriogenins and mZPC5 contain the ZP-domain and may interact to create the 3D structure of the chorion matrix.

## Conclusions

It has long been accepted that liver-expressed Chgs. are major precursor proteins of medaka chorions. In contrast, the functions of ovary-specific proteins homologous to mammalian ZPs have remained unknown. Based primarily on the mZPC5 gene expression and protein production patterns observed in the ovary, we showed that oocyte-expressed mZPC5 is one of the components of the chorion. The mZPC5 protein is incorporated into the thin layer of the chorion in previtellogenic oocytes. It may initiate further chorion formation by interacting with Chgs., possibly serving a receptor-like function for Chgs. transported from the liver. Our results suggest that during oogenesis, ovary (oocyte)-produced mZPC5 and liver-produced Chg. proteins interact to form the extracellular matrix as the chorion in medaka, *Oryzias latipes* (Fig. 9). Our studies and other researchers’ data suggest that ancient animals may have had double (liver and oocyte)- or triple (liver, oocyte, and follicle cells)-origin egg envelope proteins. As a result, different mechanisms were evolved to produce egg envelope proteins that enabled each species to survive.

## Supplementary Information

**Additional file 1:** **Supplemental Figure S1.** Diagram of the tail removal location. The photograph shows a mature medaka (Oryzias latipes) with anatomical labels for the genital pore, tail, and tail fin, as well as the location at which the tail was removed for the bleeding procedure.

## Data Availability

All data generated or analyzed during this study are included in this published article (and its supplementary information files).

## Abbreviations

BSA: Bovine serum albumin
BTTBSG: 2% BSA-Tris buffered saline containing 0.05% Tween-20 and 2% pre-immune goat serum
CBB: Coomassie brilliant blue
cDNA: Complementary deoxyribonucleic acid
Chg: Choriogenin
*chg.l*-/-: Homozygous *Choriogenin L*-knock-out medaka strain
*chg.l* + */* +: Wild-type (normal) Cab medaka
DNA: Deoxyribonucleic acid
EDTA: Ethylenediaminetetraacetic acid
EE2: 17Alpha-ethinylestradiol
ER: Estrogen receptor
FIGα: Factor in the germline alpha (transcription factor)
FSH: Follicle-stimulating hormone
HCl: Hydrogen chloride
HRP: Horseradish peroxidase
Ig: Immunoglobulin
IUPAC: International Union of Pure and Applied Chemistry
kbp: Kilobase pair
kDa: Kilodalton
MS/MS: Tandem mass spectrometry
MS-222: Tricaine methanesulfonate
MW: Molecular weight
NaCl: Sodium chloride
NaOH: Sodium hydroxide
PB: Phosphate buffer
PBS: Phosphate buffered saline
RNA: Ribonucleic acid
RT-PCR: Reverse transcription-polymerase chain reaction
SDS-PAGE: Sodium-dodecyl-sulfate polyacrylamide gel electrophoresis
STHBG: Sex steroid hormone-binding globulin
TALEN: Transcription activator-like effector nuclease
TBS: Tris buffered saline
TBSE: TBS containing 40 mM EDTA
TEA: Triethanolamine-HCl
TTBS: TBS containing 0.05% (w/v) Tween-20
ZI: Zona pellucida interna
ZP: Zona pellucida
