# Supplementary figures and images for: Medaka, Oryzias latipes, egg envelopes are created by ovarian-expressed ZP proteins and liver-expressed choriogenins
Source: Zoological Lett. 2022 Jul 28;8:11. doi: 10.1186/s40851-022-00194-2 (PMC9330664; doi:10.1186/s40851-022-00194-2)

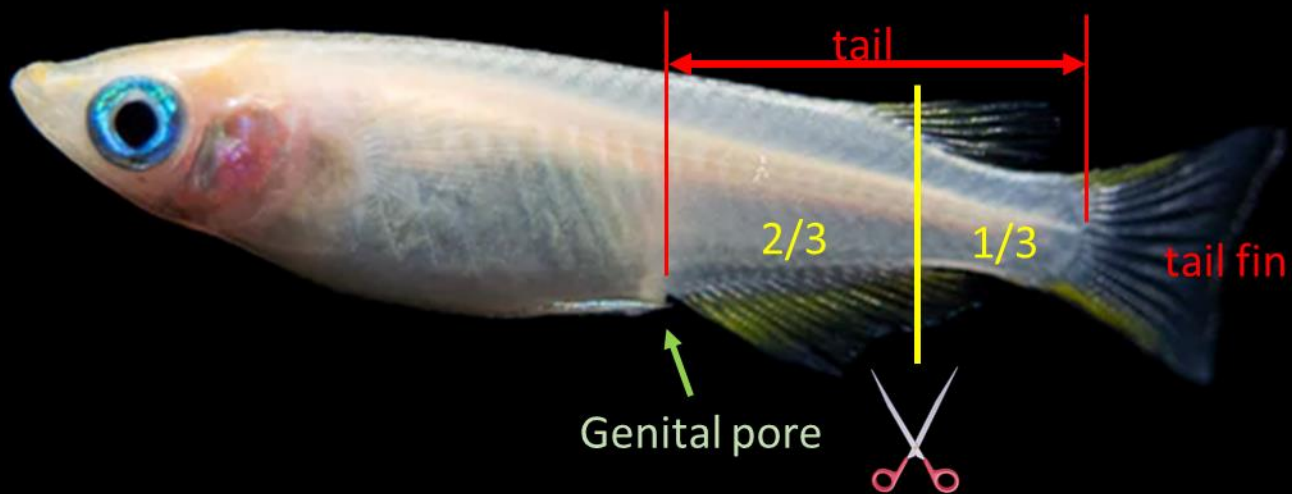

Supplement: Supplementary file 1 — Additional file 1: Supplemental Figure S1. Diagram of the tail removal location. The photograph shows a mature medaka (Oryzias latipes) with anatomical labels for the genital pore, tail, and tail fin, as well as the location at which the tail was removed for the bleeding procedure. [file 40851_2022_194_MOESM1_ESM.pdf]
